# Reply to Halpern, “Chironomid association with *Vibrio cholerae*”

**DOI:** 10.1128/spectrum.02891-23

**Published:** 2023-12-07

**Authors:** Dianshu Zhao, Afsar Ali, J. Glenn Morris, Adam Chun-Nin Wong

**Affiliations:** 1 Entomology and Nematology Department, University of Florida, Gainesville, Florida, USA; 2 Emerging Pathogens Institute, University of Florida, Gainesville, Florida, USA; 3 Department of Environmental and Global Health, College of Public Health and Health Professions, University of Florida, Gainesville, Florida, USA; 4 Genetics Institute, University of Florida, Gainesville, Florida, USA; McGill University, Sainte-Anne-de-Bellevue, Quebec, Canada

**Keywords:** *Vibrio cholerae*, chironomid, Host-microbe interactions

## REPLY

We appreciate Prof. Halpern’s interest in our work (1, 2).

We acknowledge the pioneering published work of Prof. Halpern’s lab, including the first observation made 22 years ago revealing the association between the chironomid egg masses and *Vibrio cholerae* with subsequent efforts delineating their interactions and the chironomid microbiome, some of which we have cited in our paper. To clarify, in our paper, we mentioned that the interaction dynamics between *V. cholerae* and chironomids are “largely unknown” to emphasize existing knowledge gaps and opportunities for further research, as elaborated in the Discussion section of the paper. Our intent is not to claim “nothing is known” about the subject.

Prof. Halpern made an important comment about *V. cholerae* density in the natural environment. Multiple surveys have reported that *V. cholerae* cell density in field water ranged from 10^1^ to 10^5^ cells/mL. However, we do recognize that there have been higher densities reported (>10^8^ cells/mL), particularly in water samples from cholera-endemic regions (3). Additionally, the phenomenon of *V. cholerae* entering the “viable but non-culturable state” under stressful environmental conditions, where it remains metabolically active but unable to grow on standard microbiological media, has been proposed by some researchers (4 – 7). While we remain cautious about some of these claims and findings, we acknowledge that we cannot dismiss them entirely. As explicitly stated in the paper, we speculated the dose range tested is likely higher than *V. cholerae* densities in the field, and future work will explore a broader dose range and other environmental variables such as temperature in *V. cholerae*-chironomid interactions.

**Fig 1:**
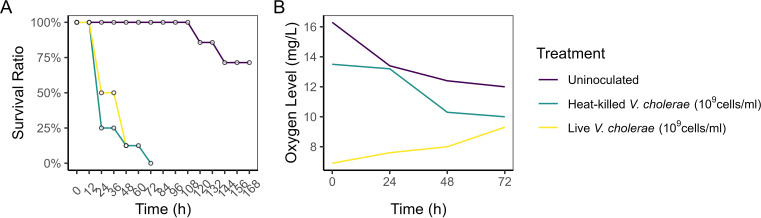
**(A**) The survival ratio of chironomid larvae exposed to heat-killed and live *V. cholerae* and the (B) oxygen level in the corresponding inoculated lake water. Chironomid fourth-stage instar larvae (eight individuals per treatment group) were exposed to dead or live *V. cholerae* at 10^9^ cells/mL using the same microcosm setup described in the manuscript. The survival ratio was monitored every 12 hours. Dissolved oxygen level in the water was tested every 24 hours using a dissolved oxygen meter (RCYAGO DO-9100).

Regarding the observation of chironomid mortality at the critical dose of 10^9^ cells/mL and Prof. Halpern’s comment about the lack of oxygen, we tested the survival of chironomid larvae when exposed to live and heat-killed *V. cholerae* at this dose. Both the live and heat-killed *V. cholerae* caused significant larval mortality (Fig. 1A), but the oxygen level in the lake water was only reduced by the live bacteria (Fig. 1B). Furthermore, in the paper, we observed no increasing hypoxia stress with rising *V. cholerae* doses. These results suggest other factors are contributing to larval death at this critically high dose. We believe that the underlying mortality mechanism warrants further investigation.

Lastly, we thank Prof. Halpern’s long-term leadership and ongoing efforts to study the relationship between *V. cholerae* and chironomids, which has laid a strong foundation to attract early-career researchers to the field. As cholera continues to be a major and growing global health concern, understanding the behavior of *V. cholerae* in the environment, including its relationship with chironomids and other natural reservoirs, is a critical component in ultimately controlling the disease. We wholeheartedly align with her vision, and we firmly believe that more research and funding should be dedicated studying of *V. cholerae*-chironomid interactions.
